# Assessing the effectiveness of a LGBT cultural competency training for oncologists: study protocol for a randomized pragmatic trial

**DOI:** 10.1186/s13063-022-06274-0

**Published:** 2022-04-15

**Authors:** Julia Seay, Eryk N. Hernandez, Jaileene Pérez-Morales, Gwendolyn P. Quinn, Matthew B. Schabath

**Affiliations:** 1 Naval Health Research Center, San Diego, CA 92152 USA; 2 Department of Cancer Epidemiology, H. Lee Moffitt Cancer Center & Research Institute, 12902 Magnolia Drive MRC-CANCONT, Tampa, FL 33612 USA; 3 Department of Obstetrics and Gynecology, Grossman School of Medicine, New York, NY 10016 USA; 4 Department of Thoracic Oncology, H. Lee Moffitt Cancer Center & Research Institute, 12902 Magnolia Drive MRC-CANCONT, Tampa, FL 33612 USA

**Keywords:** LGBT, Sexual and gender minorities, Pragmatic trial

## Abstract

**Background:**

LGBT patients may have unique psychosocial cancer care needs, and healthcare providers should have knowledge and understanding of these unique needs to effectively address disparities through the delivery of personalized healthcare. As such, our group developed and piloted a web-based LGBT cultural competency training designed specifically for oncologists called the Curriculum for Oncologists on LGBT populations to Optimize Relevance and Skills (COLORS). We designed a randomized pragmatic trial for oncologists to compare the effectiveness of the COLORS training versus a general online LGBT cultural competency training in improving LGBT-related knowledge, attitudes, and clinical practices.

**Methods/design:**

Study procedures include an 8-step approach for recruitment, randomization, retention, and completion of the interventions. Oncologists of any subspecialty who are currently practicing physicians will be identified from the American Medical Association Masterfile. Approximately 5000 oncologists will be sent a FedEx envelope with an invitation letter and study timeline. Electronic consent is obtained using a secure REDCap (Research Electronic Data Capture) portal hosted at the Moffitt Cancer Center (Tampa, FL) where the oncologists will complete the eligibility questionnaire, pre-training assessments, and then will be randomized to complete the COLORS training or an online general healthcare training offered by the National LGBT Health Education Center. Effectiveness of both trainings will be assessed utilizing self-reported measures of LGBT-related knowledge, attitudes, and affirming clinical practices. The measures will be collected before and directly after training completion, as well as 3-month post-training completion. The primary outcomes are changes in knowledge, attitudes, and practice behaviors regarding LGBT cancer patients from pre-test to post-test training in the COLORS training vs. the comparison training.

**Discussion:**

The overarching premise of this trial is to assess the effectiveness of the COLORS cultural competency training program. If successful, among oncologists who completed the COLORS training should yield statistically significantly improvements in knowledge, attitudes, and affirming practice.

## Background

The lesbian, gay, bisexual, and transgender (LGBT) community, also referred to as sexual and gender minorities, is a marginalized and medically underserved population. A growing body of evidence [1–12] demonstrates the LGBT population experiences increased risk for certain cancers, report lower satisfaction with cancer treatment, higher rates of psychological distress in survivorship, are less likely to engage in cancer early detection, and report higher rates of perceived discrimination in the health care setting. These findings suggest that LGBT patients may have unique psychosocial cancer care needs, and that healthcare providers should have knowledge and understanding of these unique needs to effectively address disparities through the delivery of personalized healthcare.

As cancer disparities in the LGBT community have largely been an ignored public health issue, consequently, there is a gap in LGBT-specific oncologist training. Despite advocacy for physician training in the unique needs of LGBT patients, there is a lack of widely adopted LGBT competency training opportunities tailored to practicing oncologists. In 2017, ASCO published guidelines [13] for oncology communities emphasizing the need for “Expanding and Promoting Cultural Competency Training and Incorporating Sexual and Gender Minority Training into Requirements and Certification Exams” and advocating for LGBT cultural competency training among oncologists specifically. Each of these recommendations acknowledge LGBT patients experience health outcomes which are directly related to the LGBT competency of their healthcare providers. For example, LGBT patients with cancer who are not comfortable discussing sexual orientation may be less likely to disclose their sexual orientation to their oncologist and are less likely to include their partner and/or family of choice in cancer care [14] which has the downstream impact of potentially increasing likelihood for poorer survivorship outcomes. Thus, these recommendations, taken together with the previous findings regarding LGBT cancer disparities, suggest oncologist education through targeted LBGT cultural competency training is crucial to improving the cancer care experiences of LGBT patients.

In 2017, our group published results [15] from a national sample of oncologists that showed oncologists have high interest in receiving LGBT health education yet have overall limited knowledge about LGBT health and cancer needs. This is problematic, as the number of people who identify as LGBT is growing, as are the number of cancer diagnoses in the USA. Based on conservative LGBT population estimates [16–18], there may be over 1 million LGBT cancer survivors living in the USA [19]. To address the interest in receiving LGBT health education and overall limited knowledge LGBT health and cancer needs, our group developed [20] and piloted [21] a web-based LGBT cultural competency training designed specifically for oncologists. Our preliminary findings [21] suggest the Curriculum for Oncologists on LGBT populations to Optimize Relevance and Skills (COLORS) training is feasible and acceptable among oncologists. Additionally, results from our pilot study showed improvements in LGBT-related knowledge and attitudes among oncologists who completed the training. Building on our formative pilot work, we designed a randomized pragmatic trial for oncologists to compare the effectiveness of the COLORS training versus a general (non-oncology focused) online LGBT cultural competency training in improving LGBT-related knowledge, attitudes, and clinical practices. Additionally, we will examine moderators of the effectiveness by potential modifiers such as age, race/ethnicity, sexual orientation and gender identity, practice setting, geographic region, and history of LGBT-related didactic training.

## Trial design

This study is a randomized pragmatic trial comparing the effectiveness of the COLORS training vs. a general healthcare (non-oncology) online LGBT cultural competency training among oncologists across the USA. Oncologists of any subspecialty who are currently practicing physicians and have not previously completed the COLORS training and/or the comparison training will be identified via the American Medical Association (AMA) Masterfile. Eligible oncologists will be randomized to complete the COLORS training or a standard online training via the National LGBT Health Education Center [22]. Effectiveness of the trainings will be assessed utilizing self-reported measures of LGBT-related knowledge, attitudes, and clinical practices. The measures will be collected before and directly after training completion, as well as 3-months post-training completion. The trial will analyze changes in LGBT-related knowledge, attitudes, and clinical practices pre- and post-training between the two study arms.

## Methods

### AMA Masterfile

The trial will identify practicing oncologists via the AMA Masterfile. The AMA Masterfile is a database of all practicing physicians in the USA independent of AMA membership or membership in any other elective organization. The AMA Masterfile is the only national database of licensed practicing physicians that does not require membership as medical students are entering into the database in the first year of school and remain in the database as long as they hold a medical license. The AMA Masterfile contains over 875,000 licensed physicians in the USA categorized by specialty and primary employment status (administrator, clinical practice, resident, etc.). All data in the Masterfile are obtained from primary sources only (medical schools, hospitals, medical societies, the National Board of Medical Examiners, state licensing agencies, etc.). The AMA Masterfile contains data on 100% of allopathic and 93% of osteopathic physicians and over 12,000 oncologists.

The trial will exclude oncologists whose primary position is listed as teaching, administrative, *locum tenen* or unclassified, as these individuals are less likely to provide clinical care to patients on a regular basis or at all. Additionally, oncologists from the state of Florida will be excluded since we conducted a pilot study [21] among oncologists at the Moffitt Cancer Center, University of Florida, and University of Miami. Residents and fellows will be excluded because they have less experience with patients and/or autonomy in determining practice protocol with patients. From a third-party vendor, the trial will obtain mailing addresses for 5000 oncologists with equal distributions across the nine US census divisions [23].

### Study procedures

**Fig. 1:**
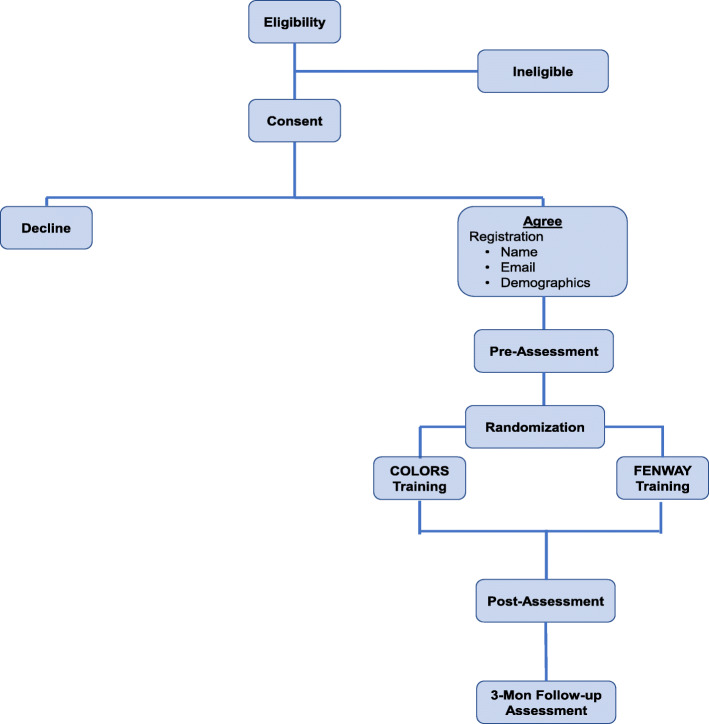
Study design and workflow to assess the effectiveness of a LGBT cultural competency training for oncologists

Study procedures include an 8-step approach as summarized in Fig. 1, for recruitment, randomization, retention, and completion of the interventions.

For step 1, approximately 5000 oncologists will be sent a FedEx envelope with an invitation letter and study timeline. The invitation includes a description of the study stating the research purpose, confidentiality of responses, the voluntary nature of the study will be explicitly stated, and a URL and QR code to enroll in the study. To increase the likelihood of response, the trial will use a modified Dillman three-wave method [24] and batched survey administration. The three-wave method includes the initial FedEx envelope and then two follow-up postcards (Table 1). Nine batches of invitations will be sent over a 14-week period with two follow-up postcards sent approximately 1 and 2 weeks, respectively, after the invitation letter. A follow-up email will also be sent among oncologists whose email addresses are available (*n* = 2,471).

**Table 1:** Timeline for invitation letter and follow-up postcards

	Invitation letter by FedEx envelope (wave 1)	Follow-up postcard 1 (wave 2)	Follow-up postcard 2 (wave 3)	Email^**1**^
**Batch 1**				
**Group 1** (*N* = 495)	2/8/2021	2/12/2021	2/26/2021	3/26/2021^1^
**Group 2** (*N* = 495)	2/15/2021	2/19/2021	3/5/2021	
**Group 3** (*N* = 495)	2/22/2021	2/26/2021	3/12/2021	
**Group 4** (*N* = 522)	3/1/2021	3/5/2021	3/19/2021	
**Group 5** (*N* = 132)	3/15/2021	3/19/2021	4/2/2021	
**Batch 2**				
**Group 6** (*N* = 709)	4/19/2021	4/27/2021	5/7/2021	5/21/2021^1^
**Group 7** (*N* = 709)	4/26/2021	4/30/2021	5/14/2021	
**Group 8** (*N* = 709)	5/3/2021	5/7/2021	5/21/2021	
**Group 9** (*N* = 722)	5/10/2021	5/14/2021	5/28/2021	

Emails were available on 2471 of the 5000 oncologists and were sent out on two separate dates for each batch of participants

For step 2, oncologists will complete an eligibility screener questionnaire on a secure REDCap (Research Electronic Data Capture) portal hosted at the Moffitt Cancer Center (Tampa, FL). Practicing oncologists who are currently seeing patients will be included in the study. Individuals whose primary position is listed as teaching, administrative, *locum tenen*, or unclassified will be excluded. Additionally, residents/fellows and those who have already completed the COLORS training and/or the comparison training will be excluded.

### Informed consent

For step 3, after eligibility is confirmed, oncologists will be automatically directed to the informed consent REDCap page. The informed consent will include the contact information for the study coordinators, such that the participants may contact the coordinators with any questions prior to signing. Participants will provide their electronic signature for the informed consent.

### Study registration

For step 4, after informed consent is provided, oncologists will be directed to the registration page to provide an email address and demographic information including age, race/ethnicity, sexual orientation, gender identity, sex assigned at birth, and geographic region.

For step 5, oncologists will be directed to complete the pre-training study measures (described below), which include measures of LGBT-related knowledge, attitudes, and practices. In the pre-training assessment, data on medical degree (MD, DO, other, or prefer not to answer) as well as on whether the participants have friends or family that identify as part of the LGBT community will also be collected. Upon completion of the pre-training measures, participants will receive a link for a $10 Amazon gift card via email.

For step 6, after completing the pre-training measures, participants are block randomized across US census division to either COLORS training or the general LGBT competency training. Randomization will occur within 24 h of completion of pre-training measures. The REDCap randomization algorithm will be used to generate the randomization. Following randomization, study participants will receive an email with instruction on how to access and complete their training assignment, and they will be reminded to complete the training within 2 weeks. Participants will complete their assigned training at their own pace, as each website allows participants to return to where they left off.

### Post-training assessments

For step 7, after oncologists complete their assigned training, they will be directed to complete the post-training measures. In addition to measuring LGBT-related knowledge, attitudes, and clinical practices, post-training measures will also gauge the feasibility and acceptability of the training. Examples of feasibility and acceptability items include “Would you recommend this course to a colleague?”, “What barriers might you have that would interfere with implementation of new information learned from this training?”, and “How can this training be improved to better impact competence, performance and/or patient outcomes?”. Following the completion of post-training measures, participants will receive an email with a link for a $20 Amazon gift card and 2 CME of MOC credits provided by the New York University.

For step 8, the three-month post-training completion, participants will be emailed a link to complete a final round of study measures to examine longitudinal changes in knowledge, attitudes, and practices. Participants will receive another $40 Amazon gift card link after they complete the 3-month follow-up measures.

### Study measures

The trial will assess effectiveness of the trainings in improving self-reported knowledge, attitudes, and clinical practice behavior using previously developed and established instruments [25–27]. Study measures will be collected at three time points: directly before the training (pre-training), directly after the training is completed (post-training), and 3-month following completion of the training.

### Knowledge measures

Consistent with the cultural awareness and knowledge dimensions of the Campinha-Bacote conceptual model [28], the knowledge measure was previously developed by the study team and collected among oncologists within the COLORS pilot study [9]. The 12-item, true/false knowledge measure (Table 2) includes general (e.g., questions pertaining sexual orientation and gender identity) and cancer-specific (e.g., questions pertaining to cancer care for LGBT) items to examine knowledge of LGBT-centric clinical and cultural competency.

**Table 2:** Knowledge questions

Question number	Question
1	Both sexual orientation and gender identity are terms that can be used to define a person’s sexual attraction to others.
2	It is important to ask your patients about both their sexual orientation and gender identity to provide them with specific resources that meet their needs.
3	To avoid confusion when treating a transgender patient, it is best to document by their biological sex pronouns in the consult notes even if they self-identify as something different.
4	All patients have similar experiences in the clinic waiting room regardless of sexual orientation and gender identity.
5	Reading material in the clinic waiting room can shape LGBT patients’ expectations about how they will be treated in the clinic.
6	Clinicians who are not LGBT can still wear a rainbow lapel pin to signal acceptance of LGBT patients.
7	LGBT people are just as likely as straight and cisgender people to have a biological family member as their primary source of social support during their cancer care.
8	If hormone therapy is not directly contra-indicated, transgender patients should continue their hormone therapy during cancer treatment, as long as they understand the risks are unknown and that they may need additional monitoring.
9	End-of-life considerations may be more complex for LGBT people, as LGBT people may be more likely to have a partner or spouse who does not have legal custody of their children.
10	It is important to build rapport with patients to indicate you are open to discuss questions not covered by standard education materials developed for heterosexual patients.
11	Standard educational materials regarding sexual side effects for cancer treatment are always easily applicable to LGBT patients.
12	It is more important to discuss body image issues with transgender cancer survivors than with lesbian, gay, and bisexual patients.

### Attitudes measures

Consistent with the cultural awareness and desire dimensions of our conceptual model, LGBT-related attitudes will be examined via the 12-item Likert-type Modern Homonegativity Scale (MHS) [29] and the Modified Attitudes Toward LGBT Patients Scale (ATLPS-M) [30]. We have modified the MHS and ATLPS-M scales to be inclusive of both sexual and gender minorities.

### Clinical practices measures

Consistent with the cultural skills and encounters dimensions of the conceptual model, LGBT-related clinical practices will be examined via 15 items from the Gay Affirmative Practice (GAP) scale [31]. This validated, Likert-type scale examines both opinions regarding which affirming practices ought to be undertaken by clinicians, as well as a self-report of one’s own LGBT-affirming and inclusive clinical practices. We have modified the GAP scale to be inclusive of both sexual and gender minorities.

### Training interventions

The experimental arm is the COLORS training, which includes both general and oncology-specific LGBT competency content. The comparison arm is composed of several web-based modules from the National LGBT Health Education Center Training website, which includes general (not oncology-specific) LGBT competency content only. The COLORS training is of comparable length (i.e., approximately 2 h) to complete as the National LGBT Health Education Center Training.

The COLORS training is a web-based training that consists of four interactive modules. Module 1 is “LGBT Basics” and focuses on SOGI terminology, intersectionality, the importance of understanding SOGI within the context of clinical care, and communication skills around discussing SOGI with patients. Module 2 is “Inclusive Environments” and focuses on the elements of the clinic environment and the messages these elements may send to LGBT patients regarding provider awareness and acceptance of LGBT patients and their support network. These elements of the environment include intake forms, non-discrimination policies, clinic decorations and reading materials, the clinic website, and clinic restrooms. In addition to calling attention to elements of the environment, this module demonstrates how oncologists can assess their own clinic environment and demonstrated actionable steps that oncologists can take to promote inclusivity and reflect that inclusivity in the clinic environment. Module 3 is “Initiating Oncology Care with LGBT Patients” and focuses on several important care considerations during the initiation of oncology care: welcoming LGBT patients’ sources of social support, making informed treatment decisions, and considerations in end-of-life discussions. Module 4 is “Issues in Cancer Survivorship among LGBT Patients” and focuses on considerations that may arise in cancer survivorship for LGBT patients, such as discussion of supportive care, body image and sexual side effects of treatment, as well as fertility concerns. Each of the modules includes a didactic portion and an interactive portion and reflect each of Campinha-Bacote’s five dimensions of cultural competency [28]: cultural awareness, cultural knowledge, cultural skill, cultural encounters, and cultural desire. The didactic portion (cultural awareness and knowledge) provides participants with module-relevant information, and the interactive portions (cultural skill, encounters, and desire) require participants to apply what they have learned to interactive case vignettes and scenarios.

The National LGBT Health Education Center Training modules are available for free online. Among participants assigned to this training will complete four modules: (1) Providing Quality Care to Lesbian, Gay, Bisexual, and Transgender Patients, (2) Affirming LGBT People through Effective Communication, (3) Achieving Health Equity for LGBT People, and (4) Improving Health Care for Transgender People. Module 1 focuses on teaching how to provide inclusive health care for LGBT patients. Module 2 focuses on how to effectively communicate with LGBT patients, including how to use affirming and inclusive language. Module 3 focuses on how to address LGBT health disparities, including how to collect and discuss information regarding sexual orientation and gender identity. Module 4 focuses on how to provide inclusive and affirming healthcare for transgender patients. Each of the modules includes didactic portions, case examples, and quizzes that serve as knowledge checks.

### Statistical analysis

The main outcomes are changes in knowledge, attitudes, and practice behaviors regarding LGBT cancer patients from pre-training to post-training assessment, as well as from pre-training to 3-month follow-up assessment (see knowledge, attitudes, and clinical practices measures listed above). We hypothesize that participants assigned to COLORS will demonstrate statistically significantly improvements in LGBT-related knowledge, attitudes, and clinical practices than participants assigned to the comparison training. Crude means with standard deviations and medians with interquartile ranges will be calculated for each measure at pre-training, post-training, and at 3-month follow-up within each study arm. Depending on the distributions of the measures, parametric or non-parametric methods, including repeated-measures analyses, will be used to test whether the measures significantly differ pre-training, post-training, and at 3-month follow-up within and between each study arm. The Holm-Bonferroni method will be used, as necessary, to adjust for multiple comparisons.

### Power calculations

Power calculations are based on the primary objective of detecting statistically significant differences between the two study arms in pre- to post-training changes for the four measures listed above. The power calculations were based on the independent main effect of measure. Each measure is calculated as a single summary score and the power calculations are based on the assumption that approximately 12% of oncologists will agree to participate and be randomized (*n* = 600) and approximately 85% of those who are randomized (*n* = 255 per arm) will complete the post-training. Assuming 255 oncologists per arm will complete the training, a 2-sided alpha of 5%, and standard deviation (SD) of 1.0, power was calculated for expected ranges of mean summary scores/scales with potential deltas of the means (0.1 to 0.4) between both arms (Table 3). For all ranges of mean scores, when the detected delta of a mean is ≥ 0.3 between the 2 arms, we have substantial power to detect statistically significant differences, modest power when delta is 0.2, and low power when delta is 0.1. If the observed SD is 1.5, power drops to 0.852, 0.616, 0.324, and 0.100 for the respective deltas. However, with the expected large sample size, we estimate the SDs to be ≤ 1.0 as found in our previous work [21]. Additionally, power substantially improves across all scenarios if the delta of the means between both arms is ≥ 5 (*not shown*). Notably, the deltas used for the power calculations were based on previous studies, as the measures used in this study are not clinical measures and thus do not have clinically significant or clinically meaningful cutoff scores or thresholds.

**Table 3:** Power calculations for the proposed scales and scores

Power for (score/scale)	Delta^a^	Mean score (control arm)	Mean score (COLORS arm)
0.994 (MHS)	0.4	12.1–12.5	12.5–12.9
0.994 (GAP)	0.4	55.1–55.5	55.5–55.9
0.922 (MHS)	0.3	12.1–12.5	12.4–12.8
0.922 (GAP)	0.3	55.1–55.5	55.4–55.8
0.615 (MHS)	0.2	12.1–12.5	12.3–12.7
0.615 (GAP)	0.2	55.1–55.5	55.7–55.7
0.203 (MHS)	0.1	12.1–12.5	12.2–12.6
0.203 (GAP)	0.1	55.1–55.5	55.2–55.6

^a^Delta of two means between both arms

## Discussion

LGBT competency trainings for physicians are known to be lacking, despite recommendations from the Institute of Medicine, The Joint Commission, and the US Department of Health and Human Services strongly advocating its incorporation into educational programs. Cultural competency is required by the Accreditation Council for Graduate Medical Education (ACGME), but a number of specialties fall short of the goal, including general surgery, internal medicine, and preventative medicine, indicating a large number of residency-trained physicians are receiving little education on this important topic [32]. Improving physician education through targeted cultural skill building is crucial. Recently, multiple national LGBT-serving organizations, including the National LGBT Cancer Network, have advocated for LGBT skill building among physicians. Some training and skill building interventions have been developed and piloted by institutions across the USA that focus on increasing LGBT-related knowledge and improving attitudes toward patients, and improving communication between patients and physicians, but their focus has been on broad cultural competency for general health and not focused on oncology care [33–35]. While most existing training modules have been shown to positively influence knowledge and attitudes toward LGBT patients, none have an exclusive oncology focus nor provide practical skills to improve practice behavior. As such, trainings are needed to meet the needs of oncologists specifically.

The current trial will determine the effectiveness of LGBT cultural competency training tailored for oncologists and will examine whether oncology-focused training significantly improves knowledge, attitudes, and clinical practices of oncologists over an existing, publicly available general healthcare LGBT cultural competency training. This trial is important as there is a gap in evidence-based LGBT-specific cultural competency training for oncologists regarding best practice behaviors across the cancer care continuum. Such training could lead to direct translational implications in reducing cancer disparities among LGBT populations.

We acknowledge the potential limitations of the current trial design. The sample is self-selected, and thus may substantially differ from those who do not elect to participate. This is important because outcomes may not be generalizable to mandatory training settings. Additionally, recruitment was limited to the AMA master file which is expansive, but lacks email information for many physicians—thus, the sample may better represent physicians who are more active in AMA. Moreover, this trial examines the effectiveness of the COLORS training among oncologists only. Thus, the findings will not be generalizable to other oncology care providers, such as nurses, residents, and fellows. Finally, while the selected outcome measures have been used in prior studies, we note these measures do not have established clinical thresholds. As such, more research is needed to determine how changes in the scores of these measures relate to meaningful changes in real-world clinical practice.

The overarching premise of this trial is to assess the effectiveness of the Curriculum for Oncologists on LGBT populations to Optimize Relevance and Skills (COLORS) cultural competency training program [20, 21]. If successful, this training should yield statistically significantly improvements in LGBT-related knowledge, attitudes, and affirming practices among oncologists and should be superior to the comparison training. In addition to assessing main effects analysis of the trial, *post hoc* analyses will be conducted to examine potential moderators of effectiveness including age, race/ethnicity, sexual orientation and gender identity, practice setting, geographic region, and history of LGBT-related didactic training.

## Trial status

Trial status: Closed

Protocol title: Developing Provider-Focused LGBT Communication Skill Building for Oncologists

Protocol version date: Version 3: March 1, 2021

Recruitment start date: February 8, 2021

Approximate end date: December 1, 2021

## Data Availability

The final dataset used and/or analyzed during the current study are available from the corresponding author on reasonable request.

## Abbreviations

COLORS: Curriculum for Oncologists on LGBT populations to Optimize Relevance and Skills
SGM: Sexual and gender minority
LGBT: Lesbian, gay, bisexual, and transgender
QOL: Quality of life
MCC: Moffitt Cancer Center
ACGME: Accreditation Council for Graduate Medical Education
AMA: American Medical Association
MHS: Modern Homonegativity Scale
GAP: Gay Affirmative Practice scale
KSM: Knowledge Summary Measure
ATLGBTP: Attitudes Toward Lesbian, Gay, Bisexual, and Transgender Patients scale
